# Bonding of TRIP-Steel/Al_2_O_3_-(3Y)-TZP Composites and (3Y)-TZP Ceramic by a Spark Plasma Sintering (SPS) Apparatus

**DOI:** 10.3390/ma9070558

**Published:** 2016-07-09

**Authors:** Aslan Miriyev, Steffen Grützner, Lutz Krüger, Sergey Kalabukhov, Nachum Frage

**Affiliations:** 1 Department of Mechanical Engineering, Columbia University, New York, NY 10027, USA; aslan.miriyev@columbia.edu; 2 Institute of Materials Engineering, TU Bergakademie Freiberg, Freiberg 09599, Germany; Steffen.Gruetzner@iwt.tu-freiberg.de (S.G.); krueger@ww.tu-freiberg.de (L.K.); 3 Department of Materials Engineering, Ben-Gurion University of the Negev, P.O. Box 653, Beer Sheva 8410501, Israel; kalabukh@bgu.ac.il

**Keywords:** fracture toughness, hardness, partially stabilized zirconia (PSZ), shear strength, solid state bonding, phase transformation, spark plasma sintering (SPS), TRIP steel, yttria-stabilized tetragonal zirconia polycrystal ((3Y)-TZP)

## Abstract

A combination of the high damage tolerance of TRIP-steel and the extremely low thermal conductivity of partially stabilized zirconia (PSZ) can provide controlled thermal-mechanical properties to sandwich-shaped composite specimens comprising these materials. Sintering the (TRIP-steel-PSZ)/PSZ sandwich in a single step is very difficult due to differences in the sintering temperature and densification kinetics of the composite and the ceramic powders. In the present study, we successfully applied a two-step approach involving separate SPS consolidation of pure (3Y)-TZP and composites containing 20 vol % TRIP-steel, 40 vol % Al_2_O_3_ and 40 vol % (3Y)-TZP ceramic phase, and subsequent diffusion joining of both sintered components in an SPS apparatus. The microstructure and properties of the sintered and bonded specimens were characterized. No defects at the interface between the TZP and the composite after joining in the 1050–1150 °C temperature range were observed. Only limited grain growth occurred during joining, while crystallite size, hardness, shear strength and the fraction of the monoclinic phase in the TZP ceramic virtually did not change. The slight increase of the TZP layer’s fracture toughness with the joining temperature was attributed to the effect of grain size on transformation toughening.

## 1. Introduction

Below 1170 °C, zirconia transforms from the tetragonal phase into a monoclinic structure, accompanied by a volume expansion of 3%–5%. In partially stabilized ZrO_2_ (by Y_2_O_3_, for instance), the tetragonal phase is metastable and displays stress-induced martensitic transformation into the monoclinic structure [1]. In the Nature manuscript named “Ceramic steel?” in 1975, the authors called partially stabilized zirconia “a ceramic analogue of steel” [2]. The toughness of zirconia realized upon such stress-induced transformation was analogously related to that observed in TRIP (transformation-induced plasticity)-steels [2,3]. TRIP-steels exhibit the phase transformation of metastable austenite into α’–martensite during plastic deformation. This phase transformation is called the TRIP-effect and depends on the chemical composition of the steel, temperature and strain rate.

Over recent decades, interest in the fabrication of metal-matrix composites based on TRIP-steels has steadily grown, due to their outstanding properties [4,5,6,7,8,9]. These steels offer a matrix material presenting a combination of suitable plasticity, high strength and reasonable energy absorption capacity for use as appropriate candidates for high mechanical load applications, such as in structural and safety automotive parts, such as crash absorbers, for instance [4,5,6,7]. The unique combination of the TRIP effect in the steel matrix and the transformation toughening of partially stabilized zirconia allows for the creation of composite materials with high damage tolerance [9]. The properties of TRIP-matrix composites with partially stabilized zirconia as a reinforcement were previously addressed [8].

For some applications, such as thermal barriers, the combination of the high damage tolerance of the composites and the extremely low thermal conductivity of pure partially stabilized zirconia may provide sandwich-shaped specimens with controlled thermal-mechanical properties. Among the possible processing techniques for the fabrication of sandwich-shaped specimens from metal and ceramic powders, spark plasma sintering (SPS) has proven to be a suitable approach. SPS is widely used for the consolidation of ceramics [10,11,12,13,14,15], metals [16,17,18], intermetallics [19,20,21] and various composites [22,23,24]. Accordingly, the properties of composites comprising a high-alloy TRIP-steel reinforced with Al_2_O_3_, Mg-PSZ or Y-TZP fabricated using SPS have been studied [8,25,26,27,28,29,30,31]. However, sintering of a (TRIP-steel-ceramic)/ceramic sandwich in a single step is very difficult due to differences in sintering temperature and the densification kinetics of the composite and the ceramic powders. On the other hand, an SPS apparatus has been successfully employed for the joining of similar and dissimilar materials [32,33,34,35,36]. The remarkable advantages of SPS joining, as compared to hot pressing and hot isostatic pressing, have been previously described [34,35]. Recently, the optimal parameters (i.e., temperature, holding time and applied pressure) for diffusion bonding of various ceramics (e.g., alumina, silicon carbide, boron carbide and magnesium aluminate spinel) using an SPS apparatus were discussed [37]. Yb- and Y-doped α-SiAlON ceramics were effectively diffusion-bonded by an SPS apparatus in less than 20 min at 1650–1700 °C. It was concluded that α-SiAlON grain growth across the joining interfaces modified the joint microstructures so as to secure high bonding strength [38]. The interaction between TRIP-steel and PSZ, particularly the interface phenomena in the TRIP-steel/PSZ system, was investigated using TEM/HRTEM and electron spectroscopy [31]. Grain boundaries between TRIP-steel and PSZ grains of the SPS-processed composite as well as the interface between the PSZ films and TRIP-steel substrate were characterized and compared. It was thus established that the deposited PSZ film was free of cracks and partly coherent with the TRIP substrate. In contrast to the thin film sample, no pronounced heteroepitaxy and/or distinct orientation relationship between TRIP-steel and PSZ grains was observed in the SPS-processed samples. Nevertheless, dislocation clusters and intersecting stacking faults were observed at the grain boundaries.

In the present study, we applied a two-step approach to obtain sandwich-shaped specimens that included separate SPS consolidation of the pure (3Y)-TZP and composites containing 20 vol % TRIP-steel, 40 vol % Al_2_O_3_ and 40 vol % (3Y)-TZP ceramic phase (hereafter, composites), and the subsequent diffusion joining of both sintered components in the same SPS apparatus. The microstructure and properties of the sintered and bonded specimens were characterized.

## 2. Results

### 2.1. Powder Consolidation in SPS

**Figure 1:**
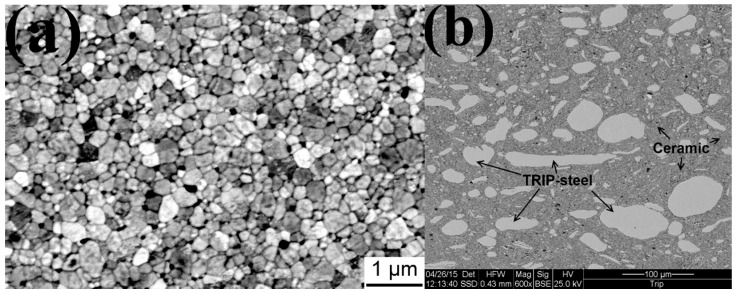
Microstructure of SPS-consolidated specimens: (**a**) (3Y)-TZP; (**b**) composite.

In Figure 1a, the microstructure of the SPS-consolidated (3Y)-TZP specimen is shown. The measured relative density of the (3Y)-TZP samples was 98.4%. Various properties of the (3Y)-TZP ceramic after SPS are shown in Table 1. The microstructure of the sintered composite is shown in Figure 1b. Bright TRIP-steel grains can be seen throughout the ceramic matrix.

### 2.2. Solid-State Joining in SPS

#### 2.2.1. (3Y)-TZP/(3Y)-TZP Joining

**Figure 2:**
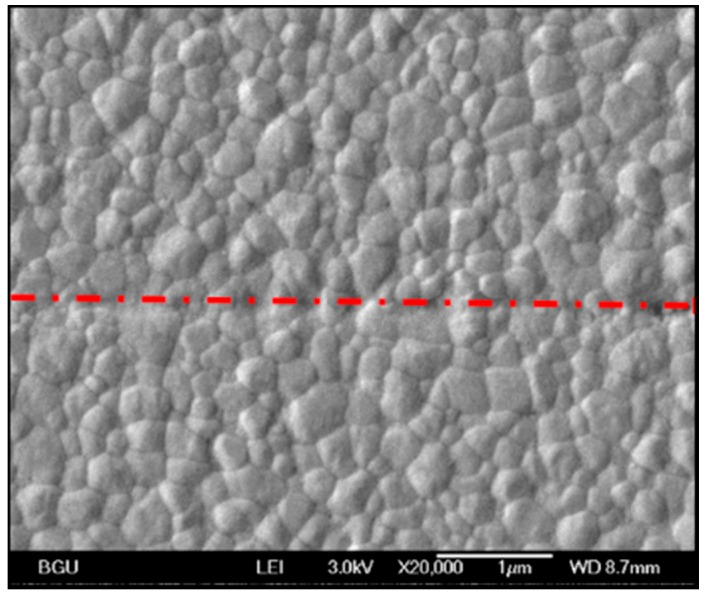
HR-SEM image of a (3Y)-TZP/(3Y)-TZP bonding area cross-section.

SPS-sintered (3Y)-TZP specimens were self-joined at 1150 °C for 120 min. The HR-SEM image of a slightly etched surface (Figure 2) shows the sub-micron equiaxed zirconia grain structure. The bonding area, marked by a dash-dot red line, is continuous, with no boundary/seam being obtained.

**Figure 3:**
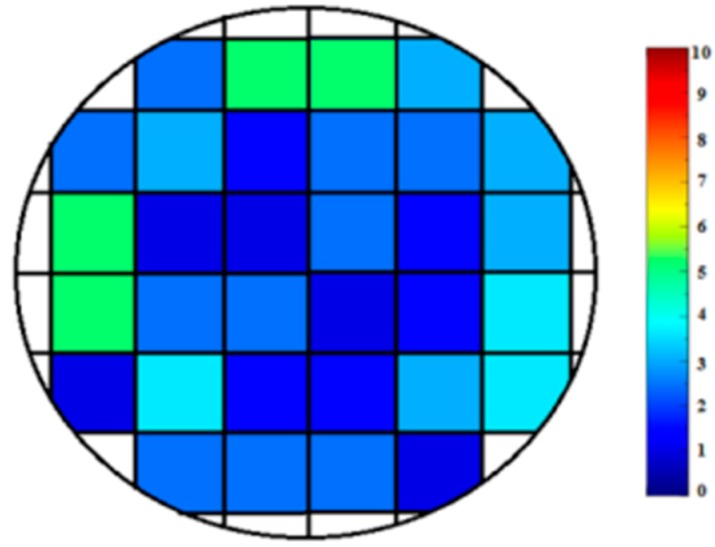
Non-destructive (ultrasonic) analysis of an SPS-joined (3Y)-TZP/(3Y)-TZP specimen. Ultrasonic wave reflections were translated into a colorimetric/numeric/scale reflecting bonding quality (0/blue: high bonding quality, 10/red: low bonding quality).

Non-destructive analysis of the joint using ultrasonic waves showed no detachments. A graphical representation of the ultrasonic test results can be seen in Figure 3. Each area unit was scanned and the reflection results were translated into a colorimetric/numeric scale, where good bonding is indicated by lower numbers (blue color on the scale) and poor bonding is indicated by higher numbers (red color on the scale). Most of the bonding area, except for some alterations at the sample periphery, showed excellent bonding quality.

#### 2.2.2. Composite/(3Y)-TZP Joining

**Figure 4:**
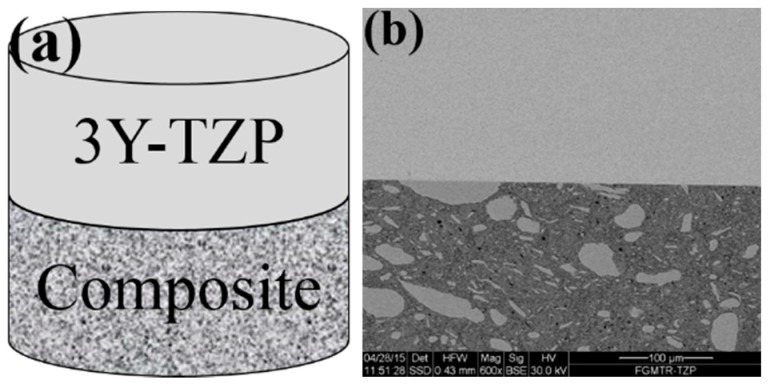
Joining a sintered composite (80 vol % ceramic) to a sintered TZP sample: (**a**) illustration; (**b**) SEM image of the bonding interface (the upper part is the (3Y)-TZP).

The main problem with the single-step sintering of steel-ceramic/ceramic sandwich structures derives from the different sintering temperatures and kinetics of the metal and ceramic components. Due to the fact that carbon from the graphite die diffuses into the steel, leading to a reduction in the melting point, the sintering temperature of the steel/ceramic composite is limited to 1150 °C. However, significantly higher temperatures (about 1350–1450 °C) are necessary to achieve fully dense (3Y)-TZP. To overcome this problem, solid-state bonding of the sintered composite with a ceramic content of 80 vol % to the sintered (3Y)-TZP part was applied (Figure 4a). No cracks or voids were observed at the bonding interface (Figure 4b).

**Figure 5:**
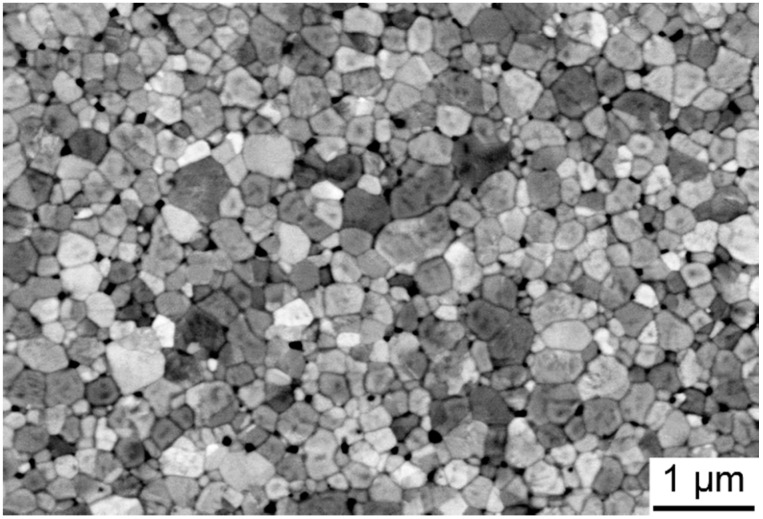
SEM image of a thermally etched surface of the (3Y)-TZP PSZ layer.

A typical microstructure of the (3Y)-TZP layer after joining for 120 min at 1150 °C is shown in Figure 5.

**Figure 6:**
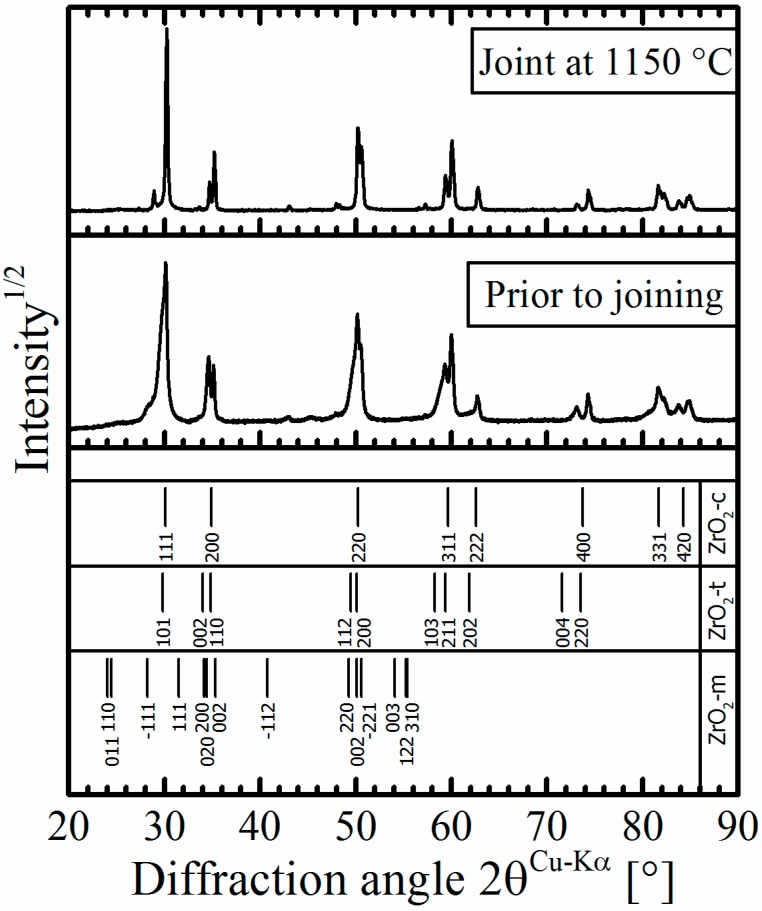
Representative XRD patterns of (3Y)-TZP taken before and after joining at 1150 °C.

Only limited grain growth (from the initial 208 nm to 226 nm after joining at 1150 °C) occurred during joining, while the crystallite size (as revealed by XRD analysis) and the fraction of the monoclinic phase in the TZP ceramic virtually did not change. This means that the martensitic phase transformation from the tetragonal to the monocline structure in the pure TZP did not occur during the joining process. The representative XRD patterns assessed before and after the joining of (3Y)-TZP are shown in Figure 6. The results are in agreement with data reported by Ruiz et al. [39], where significant grain growth and change of phase composition appeared only after isothermal heat treatment at temperatures above 1550 °C.

**Figure 7:**
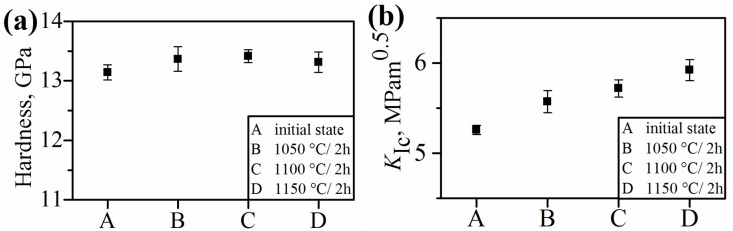
Hardness (**a**) and fracture toughness (**b**) of the TZP layer.

**Figure 8:**
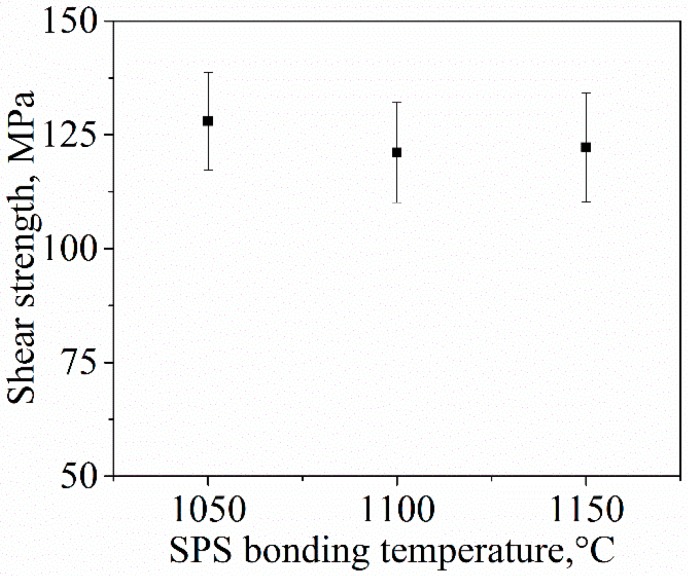
Shear strength of the SPS-bonded specimens plotted against joining temperature.

The hardness values (Figure 7a) and the shear strengths for broken SPS-bonded samples (Figure 8) likewise did not change during joining, although the fracture toughness of the TZP slightly increased with the increased joining temperature (Figure 7b). This phenomenon may be attributed to the effect of grain size on transformation toughening [40]. Above a critical grain size, tetragonal grains would spontaneously transform into the monocline structure [39,40,41,42]. However, the temperature for critical grain growth was not achieved during bonding.

## 3. Materials and Methods

### 3.1. Materials

The composite (20 vol % TRIP-steel, 40 vol % Al_2_O_3_ and 40 vol % (3Y)-TZP ceramic phase) was synthesized from high alloy austenitic CrMnNi-TRIP-steel (Table 2), alumina and yttria-stabilized zirconia powders. Median particle size of the steel powder was about 17 μm. Alumina (Pengda, Munich, Germany) and (3Y)-TZP ceramic powder (3 mole % yttria-stabilized zirconia, TOSOH, Tokyo, Japan) had median particle sizes of 0.49 µm and 40 nm, respectively.

For composite fabrication, the powders were mixed in a planetary ball mill (Pulverisette 5, Fritsch GmbH, Idar-Oberstein, Germany) under high-energy ball milling conditions for 2 h. The ball material was hardened Cr-steel, the ball diameter was 25 mm, the powder to ball ratio was 1:10 and the rotating speed was 180 rpm.

### 3.2. Processing

Specimens were fabricated in a two-step process. First, TRIP-steel/Al_2_O_3_-(3Y)-TZP composite and pure (3Y)-TZP specimens were sintered separately. The powders were consolidated as cylindrical bodies with 20 mm diameter using the SPS technique in FCT-HP D 25/2-2 apparatus. Pure (3Y)-TZP and composite samples had a final height of approximately 3 mm.

The (3Y)-TZP powder was sintered at 1400 °C under uniaxial pressure of 60 MPa for holding time of 5 min. The heating and cooling rates were 100 K/min. The (3Y)-TZP specimens were self-joined in the SPS apparatus at 1150 °C with a holding time of 120 min under an argon atmosphere (10^−2^ torr) and a uniaxial pressure of 16 MPa. The composite was consolidated at 1150 °C for a holding time of 10 min under a pressure of 60 MPa.

The mating surfaces of the samples before joining were prepared by conventional metallographic technique on a 1 μm diamond paste (Struers Nap B) stage, cleaned in acetone and dried in air. The samples were placed in a graphite die with 20 mm inner and 40 mm outer diameters and inserted into the SPS apparatus (FCT–HP D5/1 System, Rauenstein, Germany) for joining. Composite and PSZ specimens were SPS-joined at 1050, 1100 and 1150 °C, respectively, with holding time of 120 min under an argon atmosphere (10^−2^ torr) and a uniaxial pressure of 16 MPa. The temperature was measured by a pyrometer focused on the upper graphite punch. Pulse-mode DC current (pulse 5 ms and pause 2 ms) was used throughout the joining experiments. The cooling rate after bonding was about 12 K/min.

### 3.3. Characterization

Determination of the density of the samples was achieved using the Archimedes method in distilled water. Quantitative phase analysis was carried out by XRD measurement (Cu-Kα radiation). Phase compositions were estimated using the Rietveld method.

Microstructure was characterized by scanning electron microscopy (SEM). Sample surfaces were ground and polished to a 1 µm diamond finish followed by vibratory polishing for 24 h. After polishing, the prepared ceramic samples were thermally etched at 950 °C for 1 h. Subsequently, grain size and size distribution were determined by the linear intercept method on more than three SEM micrographs, such that over 900 intercepted grains were considered for each condition.

The quality of the (3Y)-TZP/(3Y)-TZP joined region was tested by ultrasonic measurement using a 15 MHz pulse/receiver probe with 3.157 mm diameter (V260, Panametrics, Houston, TX, USA). Joined specimens were divided into squares with an area of about 9 mm^2^ (3 mm × 3 mm) each. The specimens were scanned with the transducer and the resulting reflections were documented according to the known sound speed in the tested material and specimen thickness. Reflections from the bonding area and those from the bottom of the tested specimen were collected, classified qualitatively and translated according to a colorimetric scale (blue to red) and numeric scale (ranging from 0 to 10, indicating well and poor bonding quality, respectively).

**Figure 9:**
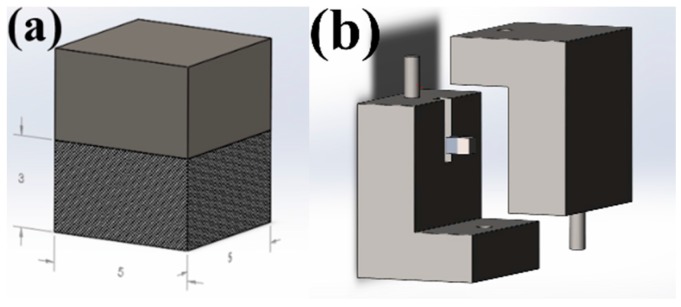

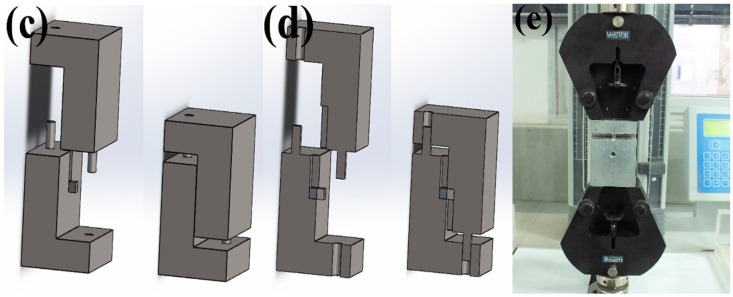
Shear test tools and specimen. (**a**) Test specimen, dimensions 5 × 5 × 6 mm^3^; (**b**) test tools and specimen; (**c**) test tools and specimen before and after mounting, full views; (**d**) test tools and specimen before and after mounting, cross-section views; (**e**) testing apparatus with mounted tools.

The maximal shear forces needed for fracture of the composite/(3Y)-TZP bonded specimens were determined using an LRX Plus apparatus (Lloyd Instruments, Fareham Hants, UK). The shear test specimen and tools are shown in Figure 9. Test specimen dimensions were 5 × 5 × 6 mm^3^ (width × length × height). The test tools consisted of the static block, into which the specimen was mounted, and the moving block. When mounted into the testing apparatus prior to testing, the static block was placed on the static portion of the apparatus, while its dynamic portion was adjusted to the moving block of the tool. Four specimens were examined for each processing parameter tested.

The fracture toughness of the (3Y)-TZP specimens after sintering and joining was investigated by the indentation method. The applied indentation load P was 98.07 N. Crack lengths were measured using an optical microscope. The fracture toughness $K_{\text{Ic}}$ (MPam^0.5^) was calculated from the equation of Niihara et al. [43] for Palmqvist cracks:

(1) $$\left(\frac{K_{\text{Ic}}\cdot \varphi }{H_{V}\cdot \sqrt{a}}\right)*{\left(\frac{H_{V}}{E\cdot \varphi }\right)}^{\frac{2}{5}}=0.035{\left(\frac{l}{a}\right)}^{-1/2}$$

where $H_{V}$ is the Vickers hardness, E is Young’s modulus, a represents the half length of the indentation diagonal, l is the mean Palmqvist crack length and φ is a pseudo-constant. The value of φ depends on the ratio between Young’s modulus and the uniaxial yield stress ($E/\sigma_{Y}$), as well as Poisson’s ratio ν; φ is reported in literature to be 2.7–3 for most ceramic materials [44]. Assuming that φ= 2.7 and Vickers hardness equals$\;H_{V}=0.4636\cdot P/a^{2}$, Equation (1) can be written as:

(2) $$K_{\text{Ic}}=0.0089\cdot {\left(\frac{E}{H_{V}}\right)}^{2/5}\cdot \frac{P}{a\cdot \sqrt{l}}$$

It should be noted that Equation (2) is applicable for Palmqvist cracks only. At low crack-to-indent ratios, the dominantly formed crack geometry are Palmqvist cracks, typically showing a ratio $\frac{l}{a}≲1.5$. At higher loads, crack geometry changes to halfpenny-shaped cracks (radial-median cracks) showing a ratio $\frac{l}{a}≳2.5$. Within the range 1.5≲l/a≲2.5, both crack systems can occur. Accordingly to the literature, Y-TZP ceramics preferentially crack in the Palmqvist rather than in the halfpenny mode [45,46].

Hardness was calculated from crack-free Vickers indentations generated at a lower applied indentation load of 4.90 N.

## 4. Conclusions

A two-stage fabrication approach was employed to obtain a sandwich-shaped specimen consisting of a high-alloy TRIP-steel/Al_2_O_3_-(3Y)-TZP composite and a (3Y)-TZP layer. The composite and ceramic specimens were separately consolidated and joined using SPS.

- No evidence of cracks or voids was observed at the composite/TZP interface.
- Limited grain growth from the initial 208 nm to 226 nm after joining at 1150 °C occurred during joining. The crystallite size and the fraction of the monoclinic phase in the Y-TZP ceramic virtually did not change.
- The hardness values and the shear force for broken SPS-bonded samples did not change during joining. Slightly increased fracture toughness of the TZP-layer with increased joining temperatures was attributed to the effect of grain size on transformation toughening.
- SPS was proven to be an effective technique for sintering and solid-state joining of ceramics and metal/ceramic composites.

## Figures and Tables

**Table 1:** Properties of the (3Y)-TZP ceramic and composite.

Property	Value
Grain size (d_50_) of (3Y)-TZP	208 nm
Grain size (d_90_) of (3Y)-TZP	339 nm
Mean crystallite size of (3Y)-TZP	60 nm
Mean crystallite size of the (3Y)-TZP phase in the composite layer	70 nm
Fraction of the monoclinic phase in (3Y)-TZP	<1 vol %
Hardness of (3Y)-TZP	13.2 GPa
Hardness of the composite	10.4 GPa
Indentation fracture toughness ($K_{\text{Ic}}$) of (3Y)-TZP	5.3 MPam^0.5^

**Table 2:** Chemical composition of CrMnNi-TRIP-steel.

Element	Fe	C	Cr	Mn	Ni	Mo	Si	S	N
Wt.%	bal.	0.031	13.50	6.49	5.73	0.07	0.48	0.007	0.034

## Abbreviations

The following abbreviations are used in this manuscript:

PSZ: partially stabilized zirconia
SPS: spark plasma sintering
TRIP: transformation-induced plasticity
TZP: tetragonal zirconia polycrystal

## References

[1] Hannink R.H.J., Kelly P.M., Muddle B.C. (2004). Transformation toughening in zirconia-containing ceramics. J. Am. Ceram. Soc..

[2] Garvie R.C., Hannink R.H., Pascoe R.T. (1975). Ceramic steel?. Nature.

[3] Gerberich W.W., Hemmings P.L., Zackay V.F., Parker E., Pratt P.L. (1969). Interactions between crack growth and strain-induced transformation. Fracture.

[4] Grässel O., Krüger L., Frommeyer G., Meyer L. (2000). High strength Fe–Mn–(Al, Si) TRIP/TWIP steels development–properties–application. Int. J. Plast..

[5] Prüger S., Kuna M., Wolf S., Krüger L. (2011). A material model for TRIP-steels and its application to a CrMnNi cast alloy. Steel Res. Int..

[6] Kovalev A., Jahn A., Weiß A., Scheller P.R. (2011). Characterization of the TRIP/TWIP effect in austenitic stainless steels using stress-temperature-transformation (STT) and deformation-temperature-transformation (DTT) diagrams. Steel Res. Int..

[7] Wolf S., Martin S., Krüger L., Martin U. (2014). Constitutive modelling of the rate dependent flow stress of cast high-alloyed metastable austenitic TRIP/TWIP steel. Mater. Sci. Eng. A.

[8] Martin S., Richter S., Decker S., Martin U., Krüger L., Rafaja D. (2011). Reinforcing mechanism of Mg-PSZ particles in highly-alloyed TRIP steel. Steel Res. Int..

[9] Aneziris C.G., Schärfl W., Biermann H., Martin U. (2009). Energy-absorbing TRIP-Steel/Mg-PSZ composite honeycomb structures based on ceramic extrusion at room temperature. Int. J. Appl. Ceram. Technol..

[10] Frage N., Kalabukhov S., Sverdlov N., Kasiyan V., Rothman A., Dariel M.P. (2012). Effect of the spark plasma sintering (SPS) parameters and LiF doping on the mechanical properties and the transparency of polycrystalline Nd-YAG. Ceram. Int..

[11] Hayun S., Kalabukhov S., Ezersky V., Dariel M.P., Frage N. (2010). Microstructural characterization of spark plasma sintered boron carbide ceramics. Ceram. Int..

[12] Hayun S., Paris V., Mitrani R., Kalabukhov S., Dariel M.P., Zaretsky E., Frage N. (2012). Microstructure and mechanical properties of silicon carbide processed by Spark Plasma Sintering (SPS). Ceram. Int..

[13] Duan R.-G., Zhan G.-D., Kuntz J.D., Kear B.H., Mukherjee A.K. (2004). Spark plasma sintering (SPS) consolidated ceramic composites from plasma-sprayed metastable Al_2_TiO_5_ powder and nano-Al_2_O_3_, TiO_2_, and MgO powders. Mater. Sci. Eng. A.

[14] Lanfant B., Leconte Y., Bonnefont G., Garnier V., Jorand Y., Le Gallet S., Pinault M., Herlin-Boime N., Bernard F., Fantozzi G. (2015). Effects of carbon and oxygen on the spark plasma sintering additive-free densification and on the mechanical properties of nanostructured SiC ceramics. J. Eur. Ceram. Soc..

[15] Poyato R., Macías-Delgado J., García-Valenzuela A., González-Romero R., Muñoz A., Domínguez-Rodríguez A. (2016). Electrical properties of reduced 3YTZP ceramics consolidated by spark plasma sintering. Ceram. Int..

[16] Niu H.Z., Chen Y.F., Zhang D.L., Zhang Y.S., Lu J.W., Zhang W., Zhang P.X. (2016). Fabrication of a powder metallurgy Ti_2_AlNb-based alloy by spark plasma sintering and associated microstructure optimization. Mater. Des..

[17] Rudinsky S., Hendrickx P., Bishop D.P., Brochu M. (2016). Spark plasma sintering and age hardening of an Al–Zn–Mg alloy powder blend. Mater. Sci. Eng. A.

[18] Minier L., le Gallet S., Grin Y., Bernard F. (2012). A comparative study of nickel and alumina sintering using spark plasma sintering (SPS). Mater. Chem. Phys..

[19] Sun Y., Vajpai S.K., Ameyama K., Ma C. (2014). Fabrication of multilayered Ti-Al intermetallics by spark plasma sintering. J. Alloys Compd..

[20] Ji G., Grosdidier T., Bernard F., Paris S., Gaffet E., Launois S. (2007). Bulk FeAl nanostructured materials obtained by spray forming and spark plasma sintering. J. Alloys Compd..

[21] Ji G., Bernard F., Launois S., Grosdidier T. (2013). Processing conditions, microstructure and mechanical properties of hetero-nanostructured ODS FeAl alloys produced by spark plasma sintering. Mater. Sci. Eng. A.

[22] Seifert M., Shen Z., Krenkel W., Motz G. (2015). Nb(Si,C,N) composite materials densified by spark plasma sintering. J. Eur. Ceram. Soc..

[23] Meir S., Kalabukhov S., Frage N., Hayun S. (2015). Mechanical properties of Al_2_O_3_/Ti composites fabricated by spark plasma sintering. Ceram. Int..

[24] Liu B.H., Su P.-J., Lee C.-H., Huang J.-L. (2013). Linking microstructure evolution and impedance behaviors in spark plasma sintered Si_3_N_4_/TiC and Si_3_N_4_/TiN ceramic nanocomposites. Ceram. Int..

[25] Decker S., Krüger L., Schneider I. Influence of steel and Mg PSZ additions on the compressive deformation behavior of an Al_2_O_3_ reinforced TRIP/TWIP-matrix-composite. Proceedings of the International Powder Metallurgy Congress and Exhibition.

[26] Krüger L., Grützner S., Decker S., Schneider I. (2015). Spark plasma sintering and strength behavior under compressive loading of Mg-PSZ/Al_2_O_3_-TRIP-steel composites. Mater. Sci. Forum.

[27] Radwan M., Nygren M., Flodström K., Esmaelzadeh S. (2011). Fabrication of crack-free SUS316L/Al_2_O_3_ functionally graded materials by spark plasma sintering. J. Mater. Sci..

[28] Decker S., Kruger L. (2015). Improved mechanical properties by high-energy milling and spark plasma sintering of a TRIP-matrix composite. J. Compos. Mater..

[29] Krüger L., Decker S., Ohser-Wiedemann R., Ehinger D., Martin S., Martin U., Seifert H.J. (2011). Strength and failure behaviour of spark plasma sintered steel-zirconia composites under compressive loading. Steel Res. Int..

[30] Decker S., Krüger L., Richter S., Martin S., Martin U. (2012). Strain-rate-dependent flow stress and failure of an Mg-PSZ reinforced TRIP matrix composite produced by spark plasma sintering. Steel Res. Int..

[31] Poklad A., Motylenko M., Klemm V., Schreiber G., Martin S., Decker S., Abendroth B., Haverkamp M., Rafaja D. (2013). Interface phenomena responsible for bonding between TRIP steel and partiallystabilised zirconia as revealed by TEM. Adv. Eng. Mater..

[32] Lee G., Yurlova M.S., Giuntini D., Grigoryev E.G., Khasanov O.L., McKittrick J., Olevsky E.A. (2015). Densification of zirconium nitride by spark plasma sintering and high voltage electric discharge consolidation: A comparative analysis. Ceram. Int..

[33] Delaizir G., Bernard-Granger G., Monnier J., Grodzki R., Kim-Hak O., Szkutnik P.-D., Soulier M., Saunier S., Goeuriot D., Rouleau O. (2012). A comparative study of spark plasma sintering (SPS), hot isostatic pressing (HIP) and microwaves sintering techniques on p-type Bi_2_Te_3_ thermoelectric properties. Mater. Res. Bull..

[34] He D., Fu Z., Wang W., Zhang J., Munir Z.A., Liu P. (2012). Temperature-gradient joining of Ti–6Al–4V alloys by pulsed electric current sintering. Mater. Sci. Eng. A.

[35] Hirose T., Shiba K., Ando M., Enoeda M., Akiba M. (2006). Joining technologies of reduced activation ferritic/martensitic steel for blanket fabrication. Fusion Eng. Des..

[36] Miriyev A., Stern A., Tuval E., Kalabukhov S., Hooper Z., Frage N. (2013). Titanium to steel joining by spark plasma sintering (SPS) technology. J. Mater. Process. Technol..

[37] Aroshas R., Kalabukhov S., Stern A., Frage N. (2015). Diffusion bonding of ceramics by spark plasma sintering (SPS) apparatus. Adv. Mater. Res..

[38] Liu L., Ye F., Zhou Y., Zhang Z., Hou Q. (2010). Fast bonding α-SiAlON ceramics by spark plasma sintering. J. Eur. Ceram. Soc..

[39] Ruiz L., Readey M.J. (1996). Effect of heat treatment on grain size, phase assemblage and mechanical properties of 3 mol % Y-TZP. J. Am. Ceram. Soc..

[40] Lange F.F. (1982). Transformation toughening. J. Mater. Sci..

[41] Bravo-Leon A., Morikawa Y., Kawahara M., Mayo M.J. (2002). Fracture toughness of nanocrystalline tetragonal zirconia with low yttria content. Acta Mater..

[42] Casellas D., Feder A., Llanes L., Anglada M. (2001). Fracture toughness and mechanical strength of Y-TZP/PSZ ceramics. Scr. Mater..

[43] Niihara K., Morena R., Hasselman D.P.H. (1982). Evaluation of K_lc_ of brittle solids by the indentation method with low crack-to-indent ratios. J. Mater. Sci. Lett..

[44] Ponton C.B., Rawlings R.D. (2013). Vickers indentation fracture toughness test Part 1 Review of literature and formulation of standardised indentation toughness equations. Mater. Sci. Technol..

[45] Kaliszewski M.S., Behrens G., Heuer A.H., Shaw M.C., Marshall D.B., Dransmanri G.W., Steinbrech R.W., Pajares A., Guiberteau F., Cumbrera F.L. (1994). Indentation Studies on Y_2_O_2_-Stabilized ZrO_2_: I, Development of Indentation-Induced Cracks. J. Am. Ceram. Soc..

[46] Cottom B.A., Mayo M.J. (1996). Fracture toughness of nanocrystalline ZrO_2_-3 mol % Y_2_O_3_ determined by vickers indentation. Scr. Mater..

